# An Aging Small-Signal Model for Degradation Prediction of Microwave Heterojunction Bipolar Transistor *S*-Parameters Based on Prior Knowledge Neural Network

**DOI:** 10.3390/mi14112023

**Published:** 2023-10-30

**Authors:** Lin Cheng, Hongliang Lu, Silu Yan, Chen Liu, Jiantao Qiao, Junjun Qi, Wei Cheng, Yimen Zhang, Yuming Zhang

**Affiliations:** 1 Key Laboratory for Wide Band Gap Semiconductor Materials and Devices of Education Ministry, School of Microelectronics, Xidian University, Xi’an 710071, China; domini_c@foxmail.com (L.C.); hllv@mail.xidian.edu.cn (H.L.); siluyan_xidian@163.com (S.Y.); 22111213679@stu.xidian.edu.cn (J.Q.); jjqi1108@163.com (J.Q.); ymzhang@xidian.edu.cn (Y.Z.); zhangym@xidian.edu.cn (Y.Z.); 2 Science and Technology on Monolithic Integrated Circuits and Modules Laboratory, Nanjing Electronic Devices Institute, Nanjing 210016, China

**Keywords:** degradation prediction, *S*-parameters, D-ELM structure, aging small-signal equivalent circuit, prior knowledge neural network

## Abstract

In this paper, an aging small-signal model for degradation prediction of microwave heterojunction bipolar transistor (HBT) *S*-parameters based on prior knowledge neural networks (PKNNs) is explored. A dual-extreme learning machine (D-ELM) structure with an adaptive genetic algorithm (AGA) optimization process is used to simulate the fresh *S*-parameters of InP HBT devices and the degradation of *S*-parameters after accelerated aging, respectively. In addition to the reliability parametric inputs of the original aging problem, the *S*-parameter degradation trend obtained from the aging small-signal equivalent circuit is used as additional information to inject into the D-ELM structure. Good agreement was achieved between measured and predicted results of the degradation of *S*-parameters within a frequency range of 0.1 to 40 GHz.

## 1. Introduction

With the rapid development of microelectronics technology, the increasing maturity of the integrated circuit process, and the continuous exploration of the depths of satellite communications, radar aerospace, and other fields, the application and development of RF semiconductor devices at microwave millimeter-wave frequencies have gradually occupied an important position in high-speed communications [1,2]. In order to reduce the cost and improve the performance, some key dimensions of the process have been continuously reduced. Accordingly, the electric field strength and current density to which the device is subjected are also increasing, which exacerbates the probability and randomness of the occurrence of various failure mechanisms and poses a serious challenge to the reliability of integrated circuits and the improvement of electronic design automation (EDA) [3].

High-reliability EDA tools need to fit, cope with, and correct real-world physical and process problems in software with a systematic approach and predictive margins as high as possible and ultimately ensure that the circuit design simulation results are consistent with the degradation results of the flow test in the reliability environment to achieve the optimal end-of-life performance/power consumption/area (EOL-PPA). The device model, as an important bridge between process and circuit design, is a key link in the DTCO flow under the new architecture, and its accuracy directly determines the accuracy and efficiency of circuit design [4]. Through the establishment of reliability models for key components in integrated systems, on the one hand, the internal physical mechanism of the device can be explored in depth, laying a foundation for the realization of the device reliability degradation simulation. On the other hand, a better prediction of the characteristic degradation can be obtained in the large-scale simulation of the electrical characteristics of circuits as well as reliability degradation effects. In addition to providing guidance for process improvement, device-level reliability models also play a key role in the reliability-aware design of integrated circuits. The establishment of efficient and accurate reliability models can be used to guide the reliability design and optimization of circuits in order to reduce the time and cost of high-reliability circuit design [5].

In recent years, research on the conventional small-signal model has focused on the characterization of non-ideal effects corresponding to ultra-high frequency rates and severe parasitics [6,7,8]. The reliability modeling-related research on this basis has been insufficient, still focusing mainly on the physical level of the device, with constraints such as long R&D cycle time, slow modeling, and incompatibility with EDA softwares [9,10]. A reliability compact model based on semi-empirical and semi-physical approaches may lack accuracy when actually characterizing degradation in time and frequency domains due to some approximations or simplifications [11,12]. Therefore, in addition to the ongoing in-depth study of the physical nature of RF microwave transistor aging and the associated reliability of compact models, we believe that there is another approach worth exploring. This method involves the use of an artificial neural network (ANN) methodology based on machine learning-assisted behavioral-level modeling [13] to achieve degradation prediction of RF devices in harsh reliability environments. The method can be trained on the degradation behavior of the device under specific reliability conditions, and then the trained network can be used to make predictions. Although the data-driven reliability-based artificial neural network model can achieve higher accuracy, it strongly relies on a large number of reliability test samples for training and learning [14]. Even if the TCAD numerical simulation tool is used to obtain the training data, a large amount of reliable experimental data is still needed to perform the complex calibration of the degradation of the amount of marching associated with aging as a time-correlated process [15]. While microwave devices involve a variety of complex combinations of conditions such as materials, geometries, frequencies, processes, etc., reliability data are more difficult to obtain and costly, and reliability experiments require strict requirements on environment, equipment, samples, etc., which require a large amount of cost investment. It can be seen that improving the traditional reliability artificial neural network model with limited reliability test samples in order to achieve efficient, accurate, and high generalization ability modeling is particularly important. Therefore, we hope to use physical/semi-physical degradation laws or formulas to guide this behavioral model and adopt a simple and effective knowledge injection to improve the reliability of the black box model.

In this paper, a prior knowledge-based neural network approach for device reliability modeling is proposed. A dual-extreme learning machine (D-ELM) is used to decompose the aging and fresh features of the device into two sub-networks to simplify the complex nonlinear mapping of the original problem. The established degradation function of the key parameters of the device is introduced into the aging equivalent-circuit model to obtain the degradation of each component of the *S*-parameters. Then, the S-parameter degradation information obtained from the coarse model is injected into the degradation sub-network as prior knowledge. In addition to the reliability parameter inputs of the original aging problem, the coarse model outputs are also used as additional inputs. At this point, the input–output mapping to be learned by the neural network is the mapping between the existing approximate reliability model outputs and the original problem, thus weakening the complex mapping relationship between reliability parameters and *S*-parameter degradation and improving the quality of the mapping. This, in turn, reduces the dependence on reliable training samples and improves accuracy with the high generalization ability of the model. Moreover, we have utilized an adaptive evolutionary algorithm to optimize the model and improve the stability of network training. Finally, the proposed PKNN-based reliability model is validated by applying it to 0.7 μm InGaAs/InP HBTs.

## 2. Device Structure and Experiment

**Figure 1:**
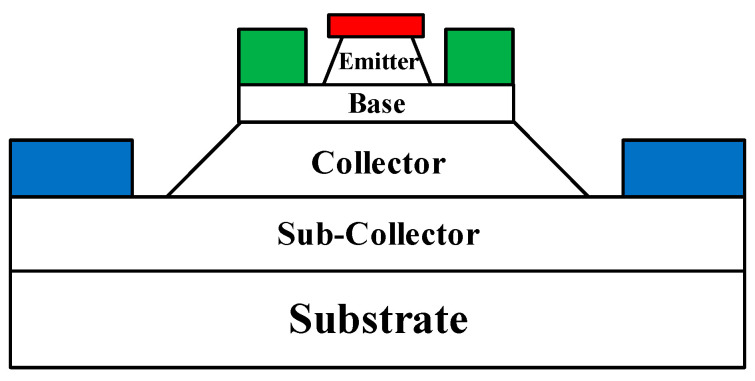
Device structure schematic.

The InP DHBT device used in this paper adopts a three mesa-structure, and its structural schematic is shown in Figure 1, in which the non-alloyed Ti/Pt/Au and Pt/Ti/Pt/Au are used for N-type and P-type ohmic contacts, respectively. Base self-alignment technology, non-contact exposure, and wet etching technology are used in the fabrication of the device.

**Figure 2:**
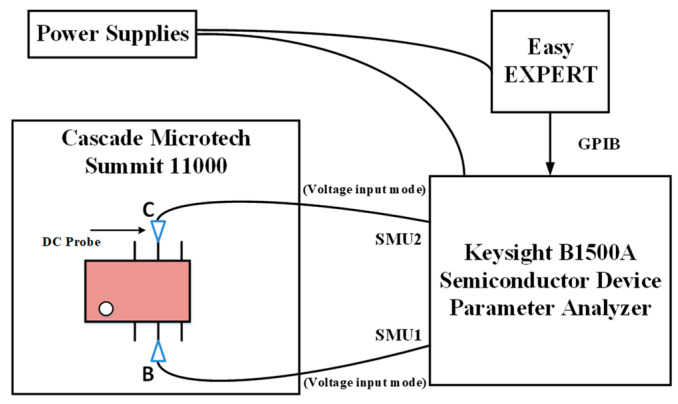
Diagram of electrical stress application.

**Figure 3:**
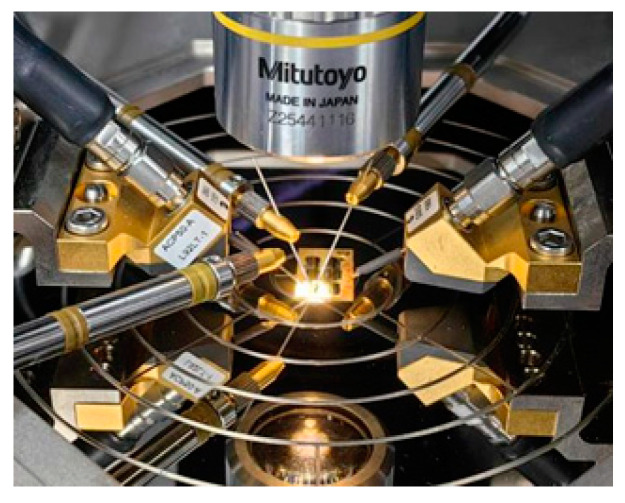
Testing equipment.

In this paper, we use a Keysight B1500A semiconductor device parameter analyzer to apply high-field electrical stress to the device by means of a DC probe. The B1500A has a very sophisticated cycling control module for DC probes to precisely apply the electrical stress, and this test module can also monitor whether the junction of the InP HBT device is broken during the test. The schematic diagram of high-field electrical stress application is shown in Figure 2, in which the base and collector of the device are connected to the SMU1 and SMU2 ports of Keysight B1500A, respectively. We set the two ports to voltage input mode and used the EasyEXPERT group+ 2015 software for the control of the electrical stress. The selection of stresses needs to be carried out before conducting the high-field stress aging test. Applying a reverse high-field stress of about 80% of the device breakdown voltage *BV*_CBO_ to the BC junction can allow the device to operate properly without breakdown and cause non-negligible degradation [16]. We have chosen about 70–90% of the breakdown voltage for high-field stress aging. The breakdown voltage of the InP HBT device used is about 4.7 V; thus, we apply constant reverse bias voltages of 3.4–4.3 V to the BC junction of the device and select four stress biases in steps of 0.3 V to perform *S*-parameter tests after up to 300 minutes of stress application while keeping the BE junction open. To accurately assess the effect of high-field electrical stress on device RF characteristics, a Rohde & Schwarz ZVA 50 Network Analyzer controlled by IC-CAP 2018 software is employed for *S*-parameter testing, and the ground–signal–ground (GSG) microwave probe was used in the on-chip measuring process. In addition, to correct the error terms introduced by the test equipment itself, a system error calibration is required before testing with VNA. In this paper, the on-chip test of InP HBT *S*-parameters is a two-port network test system, and to obtain the actual *S*-parameters of the DUT, the TOSM (Through, Open, Short, Match) system error calibration method [17,18] is used to remove the parasitic elements introduced by the equipment components such as test cables and probe tips before the reliability test. It is worth noting that the calibration of the RF on-chip test platform is performed before the electrical stress is applied. Also, attention is paid to maintaining a stable test environment during the test to ensure that the calibration conditions do not deviate. All of the above on-chip tests are performed on the CASCADE MICROTECH Summit 11000 Prober, as shown in Figure 3.

## 3. Model Technique

### 3.1. Prior Knowledge Construction

For prior knowledge neural network-based aging modeling, the first step is that we need to obtain a priori aging knowledge in the form of physical/empirical formulas or equivalent circuits, which needs to represent the degradation tendency of the device characteristics of interest but not necessarily be very accurate or complete. In this paper, we complete the construction of the prior knowledge by modeling the HBT aging equivalent circuit.

**Figure 4:**
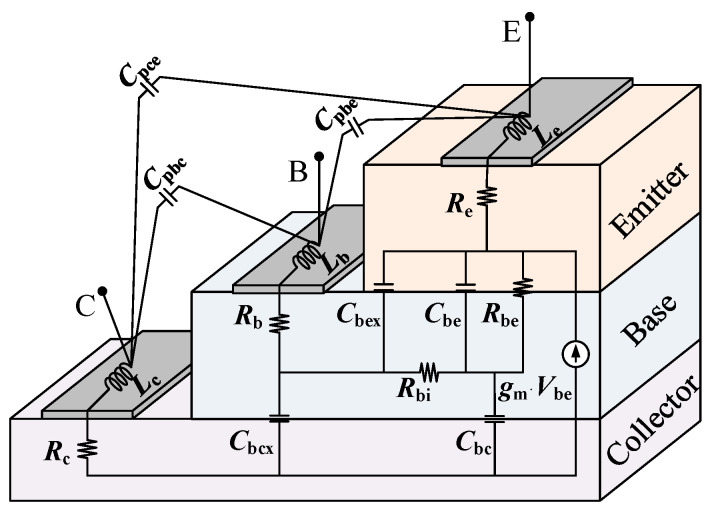
HBT small-signal equivalent circuit.

In our previous study [19], the small-signal model with base-distributed capacitances applied to HBTs was proposed, as shown in Figure 4. The equivalent circuit model consists of three modules: parasitic elements, extrinsic distributed capacitance elements, and intrinsic model elements. The outermost part of the model is the parasitic module. *C*_pbe_, *C*_pce_, and *C*_pbc_ are the base-emitter, collector-emitter, and base-collector parasitic capacitances, respectively. *L*_b_, *L*_c_, and *L*_e_ are the lead inductances associated with the base, collector, and emitter, respectively. The above pad parasitic parameters are extracted by the Open and Short Test Structure method described in [20]. *R*_b_, *R*_c_, and *R*_e_ are the series resistances associated with the base, collector, and emitter, respectively, which can be determined in the cut-off condition measurements [21]. After peeling off the parasitic elements mentioned above, the periphery of the equivalent circuit features extrinsic distributed elements, in which *C*_bcx_ is the base-collector distributed capacitance and *C*_bex_ is the base-emitter distributed capacitance. Intrinsic model elements include the dynamic base resistance *R*_bi_, the dynamic base-emitter resistance *R*_be_, the intrinsic base-emitter capacitance *C*_be_, the intrinsic base-collector capacitance *C*_bc_, the DC transconductance *G*_m0_, and the delay time *τ*. We use the peeling algorithm from our earlier work [19] to obtain the values of these extrinsic and intrinsic elements.

The aging reliability test data obtained from different batches of InP HBT devices under different stress times and stress magnitudes are characterized by the above-mentioned small-signal model topology and parameter extraction to determine the key model parameters affecting the characterization of degradation of HBT devices. Inserting a parameter Δ*P* related to the variation in the above key parameters due to aging effects, Δ*P* is defined as the difference between the key model parameters after and before degradation, which can be extracted experimentally by its variation with different stress time and stress magnitude conditions. Thus, the aging Equation (1) that can describe the degradation trend of the key parameters of the model is given. For the function establishment of the degradation amount parameter Δ*P*, we use a double exponential type function for modeling, as shown in Equation (2).

(1) $$P_{\mathrm{aging}}\left(V_{\mathrm{CB},\mathrm{stress}},t\right)=\Delta P\left(V_{\mathrm{CB},\mathrm{stress}},t\right)+P_{\mathrm{initial}}$$

(2) $$\Delta P\left(V_{\mathrm{CB},\mathrm{stress}},t\right)=A_{o}\left(1-\exp\left(\left(\exp\left(-\mu \cdot V_{\mathrm{CB},\mathrm{stress}}\right)-1\right)\cdot at\right)\right)$$

where *P*_aging_ is the value of key model parameters after degradation, Δ*P* is the amount of degradation of key model parameters, and *P*_initial_ is the value of key parameters in the fresh state. *A*_O_ is the accelerated degradation saturation factor, *a* is the degradation acceleration factor, and *μ* is the degradation acceleration index factor. The degradation equations for the above key parameters are substituted back to the model topology shown in Figure 4 to obtain the degradation trend of the *S*-parameters of the device, and we use this rough degradation law as a priori degradation knowledge to improve the quality of machine learning-assisted aging modeling mapping.

### 3.2. Dual-Extreme Learning Machine(D-ELM) Structure

ELM neural network is usually a single-hidden layer feedforward neural network. It can randomly generate the weight between the input layer and the hidden layer and the bias of the neurons in the hidden layer, and there is no need to adjust them during the training process. Only the number of neurons in the hidden layer needs to be set to obtain the unique optimal solution [22].

**Figure 5:**
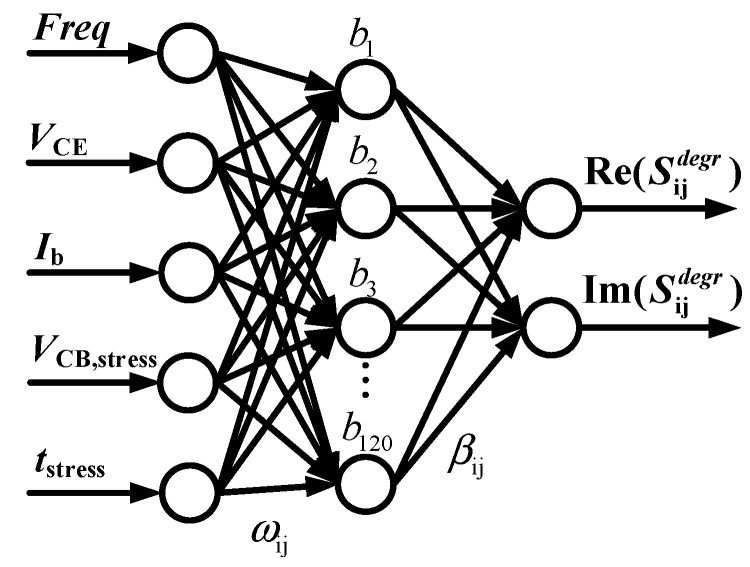
MLP structure for aging *S*-parameter modeling.

**Figure 6:**
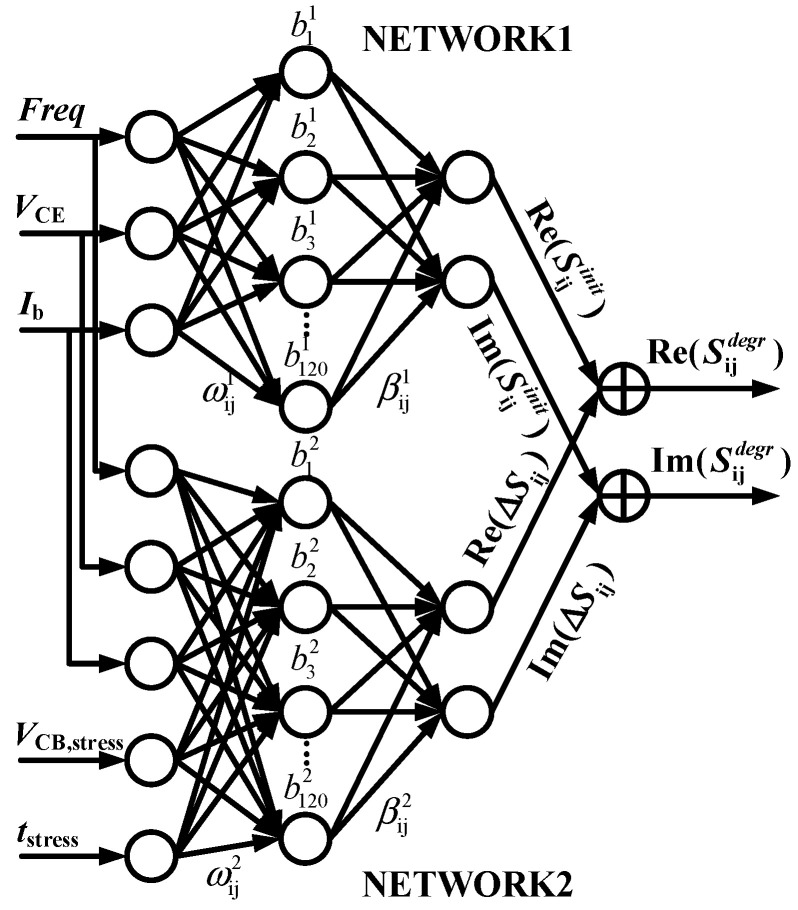
D-ELM neural network structure for aging *S*-parameter modeling.

The above traditional MLP structure for aging modeling is shown in Figure 5, which requires a large amount of degradation measurement data to construct the training set and more training time to learn the nonlinear relationships of the original problem to achieve the desired modeling accuracy. For the aging prediction of devices, therefore, we propose a D-ELM architecture, as shown in Figure 6, which decomposes the aging features and fresh features of devices into two sub-networks to simplify the complex nonlinear mapping of the original problem. NETWORK1 and NETWORK2 in Figure 6 have a common frequency input (*Freq*) and operating bias inputs (*V*_CE_, *I*_b_), and NETWORK2 has additional reliability parameter inputs (*V*_CB,stress_, *t*_stress_). Thus, we use NETWORK1 and NETWORK2 to characterize the fresh *S*-parameters and the degradation of *S*-parameters in HBTs, respectively. Then, the two sets of results are summed to obtain the aging *S*-parameters.

In addition, we give a comparison of the time consumption and accuracy of the two structures in Table 1 with the same amount of data (1340 sample sets) and the percentage of the training set (75%) for the same problem. It is evident that the proposed D-ELM architecture has a much faster training speed and modeling accuracy; it saves 17.75% training time, and the accuracy is improved by about two times compared to the traditional MLP structure.

### 3.3. PKNN-Based Aging Modeling Method

The D-ELM structure we proposed in Section 3.2 is a pure black-box model whose structure does not contain any relevant knowledge of the problem to be solved, and only the training data determine the structure of the network input–output mapping, so a large number of dataset sources are required to ensure the validity of the model.

**Figure 7:**
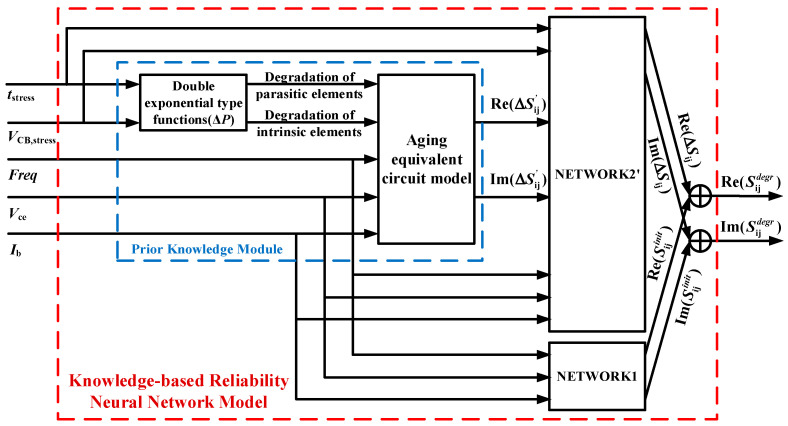
Knowledge-based reliability neural network modeling framework.

Therefore, to link the prior knowledge constructed in Section 3.1, we further improve the D-ELM structure. For the degradation network (NETWORK2), we construct the degradation formulas of the key parameters and the aging equivalent circuit as the prior knowledge module to be injected to obtain the prior knowledge-based degradation network (NETWORK2’). Subsequently, we employ this network in conjunction with NETWORK1 to simulate the *S*-parameters of the device in the fresh state to obtain the knowledge-based reliability neural network model, whose modeling framework is shown in Figure 7. The mathematical derivation process is shown in the following equations:

For NETWORK1 and NETWORK2’, their input vectors are shown in Equations (3) and (4), respectively:

(3) $$X_{q}^{N1}={\left[{\mathrm{Freq}}_{q},V_{ce,q},I_{b,q}\right]}^{T}{,}_{}(q=1,2,\cdots ,T1)$$

(4) XqN2=Freqq,Vce,q,Ib,q,VCB,stress,q,tstress,q,Re(ΔSij′)q,Im(ΔSij′)qT,(q=1,⋯,T2)

where *N*1 and *N*2 represent NETWORK1 and NETWORK2’, respectively. T1 and T2 are the number of training set samples for NETWORK1 and NETWORK2’, respectively. Δ*S*_ij_′ is the output of the coarse model degradation obtained from the aging equivalent circuit. The corresponding training target matrices of the two networks are shown in Equations (5) and (6) below:

(5) TN1=ReSijinit1ReSijinit2⋯ReSijinitT1ImSijinit1ImSijinit2⋯ImSijinitT18×T1

(6) TN2=ReΔSij1ReΔSij2⋯ReΔSijT2ImΔSij1ImΔSij2⋯ImΔSijT28×T2

where *S*_ij_^init^ is the value of *S*-parameters in the fresh state. *S*_ij_^degr^ is the value of *S*-parameters after degradation.

In addition, the connection weight vectors between the input and hidden layers of NETWORK1 and NETWORK2’ are shown in Equations (7) and (8), and the bias vectors of the hidden neurons are shown in Equations (9) and (10). They can be randomly assigned before training and remain unchanged during the training process.

(7) $$W_{p}^{N1}=\left[\omega_{p1}^{N1},\omega_{p2}^{N1},\omega_{p3}^{N1}\right]{,}_{}(p=1,2,\cdots ,H1)$$

(8) $$W_{p}^{N2}=\left[\omega_{p1}^{N2},\omega_{p2}^{N2},\cdots ,\omega_{p13}^{N2}\right]{,}_{}(p=1,2,\cdots ,H2)$$

(9) $$B^{N1}={\left[b_{1}^{N1},b_{2}^{N1},\cdots ,b_{H1}^{N1}\right]}^{T}$$

(10) $$B^{N2}={\left[b_{1}^{N2},b_{2}^{N2},\cdots ,b_{H2}^{N2}\right]}^{T}$$

where H1 and H2 represent the number of hidden layer neurons in NETWORK1 and NETWORK2’, respectively. The transfer function of the hidden layer neuron is

(11) $$g(\gamma )=\frac{1}{1+e^{-\gamma }}$$

where *γ* is the weighted sum of all inputs to the neuron. 

After all the functional variables in the neural network algorithm are defined, we can obtain the hidden layer output matrix as shown in Equations (12) and (13).

(12) $$H^{N1}={\left[\begin{array}{cccc} g(W_{1}^{N1}X_{1}^{N1}{+b}_{1}^{N1}) & g(W_{2}^{N1}X_{1}^{N1}{+b}_{2}^{N1}) & \cdots & g(W_{H1}^{N1}X_{1}^{N1}{+b}_{H1}^{N1})\\ g(W_{1}^{N1}X_{2}^{N1}{+b}_{1}^{N1}) & g(W_{2}^{N1}X_{2}^{N1}{+b}_{2}^{N1}) & \cdots & g(W_{H1}^{N1}X_{2}^{N1}{+b}_{H1}^{N1})\\ \vdots & \vdots & \ddots & \vdots \\ g(W_{1}^{N1}X_{T1}^{N1}{+b}_{1}^{N1}) & g(W_{2}^{N1}X_{T1}^{N1}{+b}_{2}^{N1}) & \cdots & g(W_{H1}^{N1}X_{T1}^{N1}{+b}_{H1}^{N1}) \end{array}\right]}_{T1\times H1}$$

(13) $$H^{N2}={\left[\begin{array}{cccc} g(W_{1}^{N2}X_{1}^{N2}{+b}_{1}^{N2}) & g(W_{2}^{N2}X_{1}^{N2}{+b}_{2}^{N2}) & \cdots & g(W_{H2}^{N2}X_{1}^{N2}{+b}_{H2}^{N2})\\ g(W_{1}^{N2}X_{2}^{N2}{+b}_{1}^{N2}) & g(W_{2}^{N2}X_{2}^{N2}{+b}_{2}^{N2}) & \cdots & g(W_{H2}^{N2}X_{2}^{N2}{+b}_{H2}^{N2})\\ \vdots & \vdots & \ddots & \vdots \\ g(W_{1}^{N2}X_{T2}^{N2}{+b}_{1}^{N2}) & g(W_{2}^{N2}X_{T2}^{N2}{+b}_{2}^{N2}) & \cdots & g(W_{H2}^{N2}X_{T2}^{N2}{+b}_{H2}^{N2}) \end{array}\right]}_{T2\times H2}$$

In turn, the output matrices of the two networks are obtained separately:

(14) $$\overset{\wedge }{T^{N1}}=f(\mathrm{Freq},V_{ce},I_{b})=H^{N1}\beta^{N1}$$

(15) TN2∧=f(Freq,Vce,Ib,VCB,stress,tstress,Re(ΔSij′),Im(ΔSij′))=HN2βN2

where *β*^*N*1^ and *β*^*N*2^ are the connection weight matrices between the hidden and output layers to be solved during the neural network training, respectively. The optimal weight between the hidden and output layers can be found by obtaining the Moore–Penrose generalized inverse [23] of the output matrix of the hidden layer to complete the training of the ELM network. In summary, we can obtain the objective function of the proposed PKNN-based D-ELM network training as shown in Equation (16), and its least squares solution can be obtained from Equation (17).

(16) $$\min(||\overset{\wedge }{T^{m}}-T^{m}{\left|\right|}^{2})=\min({\left||H^{m}\beta^{m}-T^{m}|\right|}^{2}){,}_{}(m=N1\;\mathrm{or}\;N2)$$

with

(17) $$\overset{\wedge }{\beta^{m}}={\left(H^{m}\right)}^{†}T^{m}$$

At this point, the network structure, corresponding neuron weights, and bias of the networks are determined. In addition to the reliability parametric inputs of the original problem, the *S*-parameter degradation information obtained from the aging small-signal equivalent circuit is used as an additional input to NETWORK2’ in the constructed knowledge-based reliability neural network model. Then, the input–output mapping to be learned by the neural network is the mapping between the output of the existing approximate reliability model and the original problem, which provides the degradation knowledge to weaken the strong nonlinear relationship between the reliability coefficients and the degradation of *S*-parameters and improve the quality of the mapping. This, in turn, serves to reduce the training dataset requirement and improve the network generalization capability and modeling efficiency.

### 3.4. Model Optimization

For traditional ELM networks, random selectivity makes the accuracy and convergence of neural networks relatively unstable. Typically, a network needs to be initialized many times to try out the optimal value. The adaptive genetic algorithm (AGA) is an evolutionary algorithm, a method to search for the optimal solution by simulating the natural evolutionary process [24]. Therefore, we use the AGA to automatically optimize the input weights and hidden layer bias of the networks to avoid the effects of random initialization.

**Figure 8:**
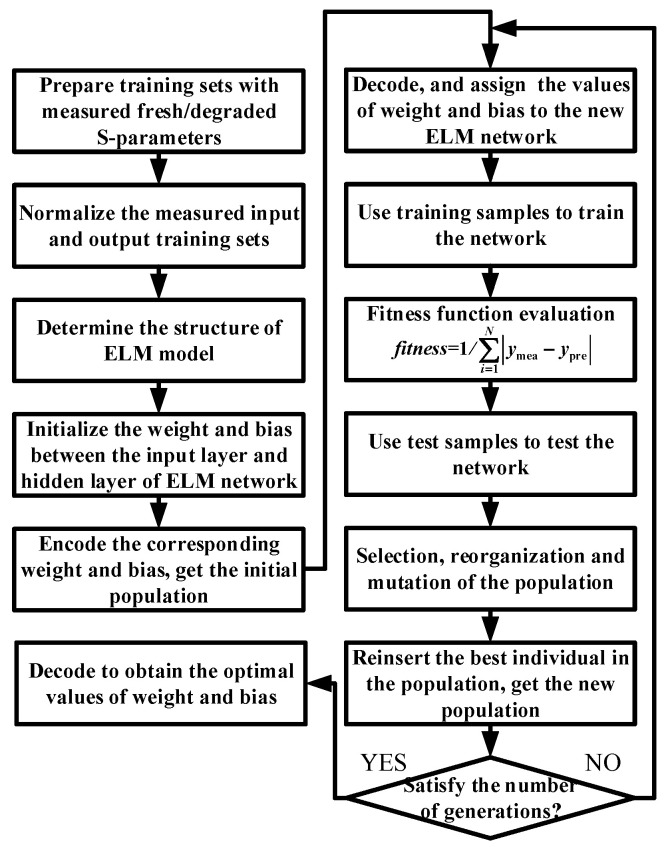
Flowchart of AGA optimization.

This optimization process is described in the flowchart shown in Figure 8. First, we need to prepare the training sample sets of input and output data with measured fresh/degraded *S*-parameters for NETWORK1 and NETWORK2’. After normalizing the above training data, the connection weight between the input and hidden layers and the bias of the neurons in the hidden layer are randomly initialized in the D-ELM network shown in Figure 6. Since the AGA cannot directly deal with the parameters of the problem space, they need to be represented as individuals in the genetic space by coding. A fitness evaluation is then performed for each individual. The fitness function is expressed by Equation (18).

(18) $$\mathrm{fitness}=1/\sum_{i=1}^{N}\left|y_{mea}-y_{pre}\right|$$

where *y*_mea_ and *y*_pre_ represent the measured output and the predicted output of the neural network, respectively. *N* is the total number of training data.

After the fitness evaluation, 15% of individuals in the paternal generation will be rejected, and the remaining individuals will undergo reorganization and mutation to obtain offspring. The corresponding reorganization probabilities *p*_x_ and mutation probabilities *p*_m_ are set to be adaptive, as shown in Equations (19) and (20):

(19) $$p_{x}=\left\{\begin{array}{c} k_{1}(f_{\max}-f)/(f_{\max}-f_{avg}),f\geq f_{avg}\\ k_{2},{f\;<\;f}_{avg} \end{array}\right.$$

(20) $$p_{m}=\left\{\begin{array}{c} k_{3}(f_{\max}-f^{\prime })/(f_{\max}-f_{avg}),f^{\prime }\geq f_{avg}\\ k_{4},f^{\prime }{\;<\;f}_{avg} \end{array}\right.$$

where *f*_max_ is the maximum fitness in the population. *f*_avg_ is the average fitness of the population. *f*_max_–*f*_avg_ reflects the convergence state of optimization. *f* is the larger fitness of the two individuals to be reorganized. *f*^′^ is the fitness of the individual to be mutated. *k*_1_, *k*_2_, *k*_3,_ and *k*_4_ are constants less than 1.

For high-quality individuals with high fitness, *p*_x_ and *p*_m_ are reduced to preserve them; for low-quality individuals with low fitness, *p*_x_ and *p*_m_ are increased to eliminate them. In addition, in the early stage of iterative optimization, the population needs a large reorganization and mutation probability to achieve a fast search for the optimal solution, while in the late stage of convergence, the population needs a small reorganization and mutation probability to enable the population to converge quickly after searching for the optimal solution. Then, the superior individuals in the offspring were reinserted into the paternal generation to form a new population. The above process will not stop until the number of training cycles satisfies the number of evolutionary generations set in the experiment. Finally, we decode to obtain the optimal values of the weight between the input layer and the hidden layer and the bias of the hidden neurons. At this point, we can obtain the trained AGA-D-ELM neural network model to predict the aging *S*-parameters.

## 4. Results and Discussion 

**Figure 9:**
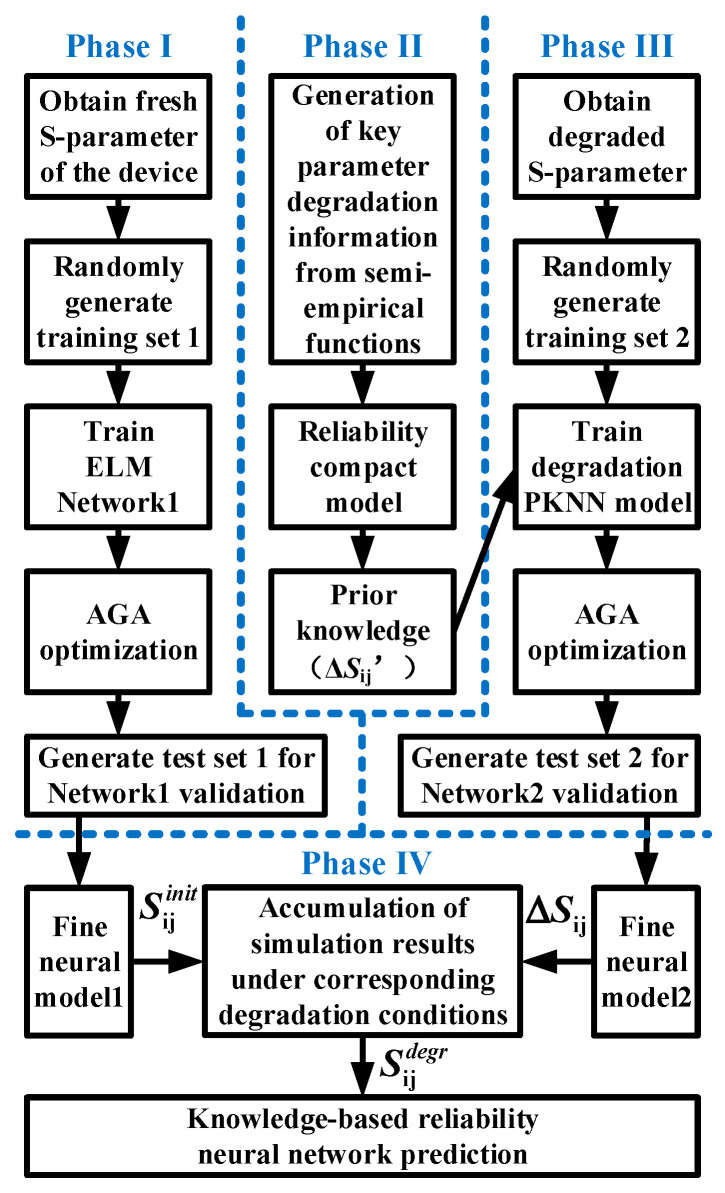
Flowchart of the proposed PKNN-based aging modeling method.

In order to more intuitively represent the modeling process in this paper, we use the PKNN-based aging small-signal modeling flowchart shown in Figure 9 to briefly summarize the above steps of device small-signal aging knowledge construction, knowledge injection, D-ELM network training, and network optimization. In the first stage, we use NETWORK1 to train and predict the *S*-parameter samples in the fresh state of the device. Then, we transform the degradation trend of the key parameters into the degradation information of the *S*-parameters after applying stress to the device through the aging compact model in the second stage. In the third stage, we inject the rough aging information obtained in the second stage into the degradation NETWORK2, then train and optimize this network. Finally, the aging *S*-parameters of the device are obtained by accumulating the predicted outputs of the two networks under the corresponding degradation conditions. The proposed aging modeling technique is evaluated and validated by modeling the aging *S*-parameters of different batches of InP HBTs degraded under different stress magnitudes and times in the frequency range of 0.1–40 GHz.

Trained artificial neural network models are usually evaluated using the prediction error of the test sample sets. For the reliability of the ANN model in this paper, the main factors affecting its network training in terms of dataset sample requirements are the total reliability sample size and the percentage of the training sample sets. Therefore, we set up seven groups of training experiments, as summarized in Table 2, to verify the validity and accuracy of the proposed model. The first, second, and third groups keep the same total sample size (2412 sets) and 50% training set share. These three experiments were conducted to model the degradation of each component of the *S*-parameters using the traditional ELM structure and the proposed PKNN structure, respectively, to compare the degradation prediction ability of the two networks under the same sample size and the same training conditions with different amounts of knowledge injection. Additionally, to further illustrate the superiority of the aging modeling approach proposed in the paper, keeping the same conditions as the first three sets of experiments, the widely used backpropagation (BP) and radial basis function (RBF) algorithms are applied for additional comparison in the fourth and fifth groups, respectively. Then, we set up a sixth set of experiments, still keeping the same total sample size (2412 sets) as the first five sets of experiments but randomly selecting 80% of the total sample size to train the network on the traditional ELM structure. This was conducted to compare the results with the second and third sets of experiments on prediction with the proposed PKNN structure to verify the prediction ability of the proposed model after the dependence on the reliability training samples is reduced. Finally, we supplemented a seventh set of experiments by taking about 60% (1458 sets) of the total sample size of the first six sets, with the training set share remaining the same as that of the sixth set. Then, we compared it with the prediction results of the ELM structure in the sixth set of experiments to further compare and illustrate the prediction ability of the proposed PKNN model with the reduction in the total reliability sample size. In addition, after keeping the same total sample size and fixing the training set share for the third and sixth sets of experiments, the test sets they used for validation also naturally formed a set of control groups with a share of 50% and 20%, respectively. The results of the comparison between these two sets of experiments can be used to further illustrate how well the generalization ability of both the traditional ELM structure and the proposed PKNN structure is. In Table 3, we give the mean relative errors of the prediction of the degradation of each component of the *S*-parameters in the seven sets of experimental test sets, and their error equations are shown below.

(21) $$\mathrm{MRE}=\frac{100\%}{N}\cdot \sum_{i=1}^{N}\left|\frac{y_{mea}-y_{pre}}{y_{mea}}\right|$$

where *y*_mea_ and *y*_pre_ represent the measured output and the predicted output of the neural network, respectively. *N* is the number of data.

From the comparison of the prediction results between the first and third sets of experiments, the overall prediction accuracy of the PKNN model for the degradation of each component of the *S*-parameter is better than that of the ELM structure. The improvement is at least 51.6% for each component and a reduction of 74.3% in the overall error for the same sample size and training configuration. For the fourth and fifth groups of BP and RBF algorithm networks, the overall prediction accuracy of PKNN is about three times higher than theirs. From the comparison of the prediction results between the third and sixth sets of experiments, compared with the aging prediction of the ELM structure without knowledge injection, the PKNN-based aging model can meet or even exceed its prediction accuracy for all the components of the *S*-parameters with at least a 37.5% reduction in the amount of training data. Furthermore, for the second set of experiments with only 50% knowledge injection, although the accuracy of the PKNN model decreased compared to the third set, the total prediction accuracy still improved by 11.4% compared to the sixth set. Moreover, the size of the test samples to be validated for the PKNN model in the third set of experiments (1206 sets) is about 2.5 times larger than that of the ELM model in the sixth set of experiments (482 sets), while its overall error has been reduced by 57.2%, which is evident in the significant improvement of its generalization ability. From the comparison of the prediction results between the sixth and seventh sets of experiments, the proposed PKNN model reduces its overall error by 63.2%, with the total reliability sample size reduced by at least 39.6% while controlling the same occupancy of the training set, further demonstrating the superiority of the proposed model in terms of reliability sample dependency while guaranteeing accuracy.

**Figure 10:**
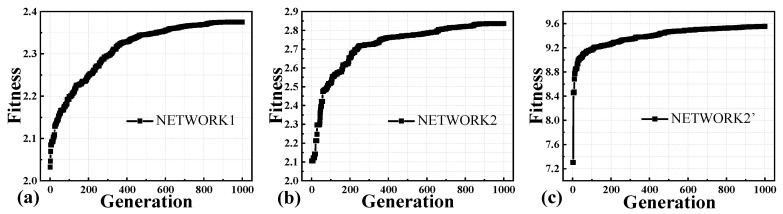
Evolution of fitness during optimization for (**a**) NETWORK1, (**b**) NETWORK2, and (**c**) NETWORK2’.

In addition, we have further compared the third and sixth sets of experiments, which have been verified to have improved prediction accuracy and generalization ability in terms of the efficiency and convergence of the network training optimization. In this paper, for each set of training samples, the input layer of NETWORK1, which describes the S-parameters of the fresh state of the device, has three input neurons, and the input layers of NETWORK2 and NETWORK2’, which describe the amount of degradation of the S-parameters of the device, have five and thirteen input neurons, respectively. The D-ELM structure is a single hidden layer structure with 120 hidden neurons. Therefore, the number of individuals to be optimized in the AGA optimization process is 480 for the population of NETWORK1 and 720 and 1680 for the population of NETWORK2 and NETWORK2’, respectively. The stopping criterion is set to a maximum of 1000 generations. The fitness evolution curve of NETWORK1 during the training iterations is shown in Figure 10a. The total optimization time is 1956, and its fitness value is optimized by 17.2%. For NETWORK2 using the ELM structure and NETWORK2’ using the PKNN structure, their fitness evolution curves are shown in Figure 10b,c. The optimized convergence duration is 4939s and 2173s, respectively, and the convergence speed is accelerated by about 56.0%, and their fitness values are optimized by 34.6% and 30.8%, respectively. The results of the above networks in terms of efficiency and convergence of training and optimization are summarized in Table 4, from which it can also be seen that the overall fitness range of the PKNN aging model for its population during optimization iterations is about three times higher than that of the traditional ELM network. The high superiority of individuals throughout the optimization period further illustrates the advantages of the proposed model in terms of simulation accuracy.

**Figure 11:**
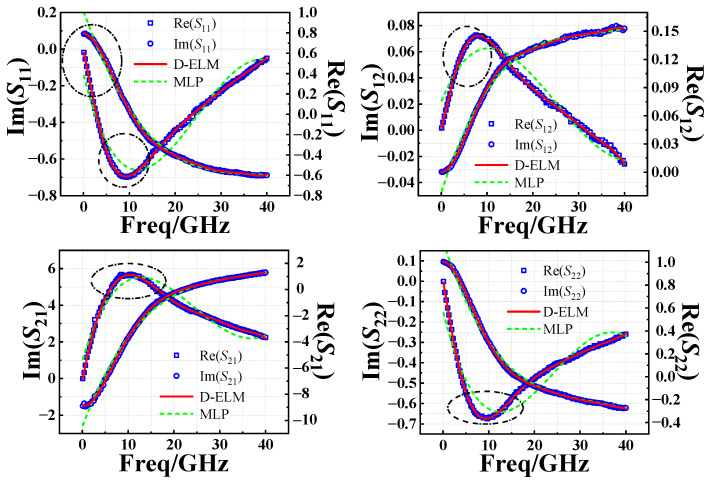
Comparison between predicted and measured results of the S-parameters in the fresh state from 0.1 to 40 GHz.

**Figure 12:**
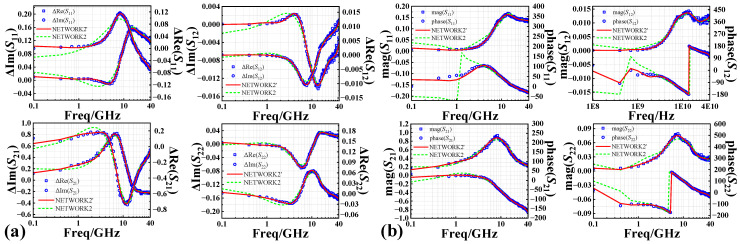
Comparison between predicted and measured results of (**a**) real and imaginary parts and (**b**) magnitude and phase of degradation of S-parameters from 0.1 to 40 GHz at *V*_CB,stress_ = 3.4 V, *t*_stress_ = 250 min.

**Figure 13:**
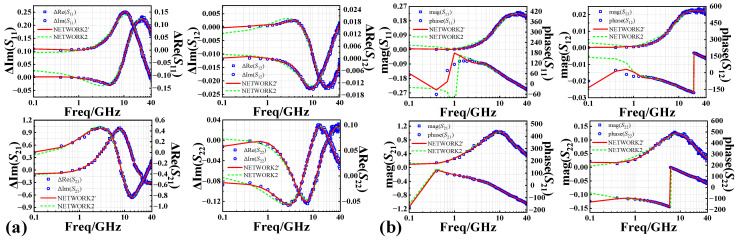
Comparison between predicted and measured results of (**a**) real and imaginary parts and (**b**) magnitude and phase of degradation of S-parameters from 0.1 to 40 GHz at *V*_CB,stress_ = 4.3 V, *t*_stress_ = 310 min.

Using the prior knowledge-based AGA-D-ELM network developed above, we predict the *S*-parameters of InP DHBT devices in the fresh state and the degradation of the *S*-parameters under different stress magnitudes and stress times. A comparison of the simulations of NETWORK1 and the conventional MLP network for the fresh *S*-parameters is given in Figure 11, which further illustrates the accuracy of the predictions of the D-ELM structure. As shown in Figure 12a and Figure 13a, we observed the performance of different batches of 0.7 × 13 μm^2^ InP DHBT devices under the bias of (*V*_CE_ = 1.6 V, *I*_b_ = 200 μA) and subjected to two stress conditions: (*V*_CB,stress_ = 3.4 V, *t*_stress_ = 250 min) and (*V*_CB,stress_ = 4.3 V, *t*_stress_ = 310 min). The model predictions of the degradation of the real and imaginary parts of each component of *S*-parameters are compared with the corresponding test results using the NETWORK2’ and NETWORK2 trained in the second and third sets of experiments, respectively. In addition, to further complement the predictive effectiveness of the proposed model, we present the simulation results of the model in Figure 12b and Figure 13b for the magnitude and phase of the degradation of each *S*-parameter component in the Advanced Design System (ADS). The simulation accuracy of NETWORK2’ on the degradation of S-parameters is still able to reach or even be better than that of NETWORK2 after the dependence on reliability training samples is reduced. It can be seen that the proposed PKNN network structure is able to predict the degradation trend of the *S*-parameters of the HBT devices after the accelerated aging well, which provides guidance for the establishment of a highly efficient and accurate aging model for microwave devices in practical applications.

## 5. Conclusions

A prior knowledge-based aging small-signal neural network model is proposed for the degradation prediction of *S*-parameters of microwave HBTs. The adopted dual-extreme learning machine architecture decomposes the aging features and the fresh features of the device into two sub-networks, respectively, to simplify the complex nonlinear mapping of the original problem. Moreover, we transform the degradation trend of the device key parameters into the degradation information of the *S*-parameters after the device is stressed by the aging compact model. Compared with the traditional MLP degradation sub-network architecture, the proposed knowledge-based reliability neural network model incorporates all this degradation information. This integration improves prediction accuracy with reduced reliability training sample dependency, as well as improving the generalization ability of the network. The superiority of the convergence efficiency of the proposed model in the optimization process is further verified by introducing an adaptive genetic algorithm to automatically optimize the input weights and hidden layer biases in the network to circumvent the instability caused by random initialization. Finally, the validity and accuracy of the proposed PKNN-based aging model are verified by comparing the predicted and measured results of the *S*-parameter degradation of different batches of 0.7 × 13 μm^2^ InGaAs/InP DHBTs in the frequency range of 0.1–40 GHz.

## Figures and Tables

**Table 1:** Comparison of time consumption and accuracy of MLP and D-ELM structures.

Network Types	Time Required Per Training Session(s)	Residual Error (%)
MLP	11.72	8.22
D-ELM	9.64	3.93

**Table 2:** Experimental setup of the dataset for training and validation.

Group	Training Networks	Knowledge Samples	Knowledge Injection Share (%)	Total Sample Size	Training Data Share (%)	Training Sample Sets	Test Sample Sets
1	ELM	0	0	2412	50	1206	1206
2	PKNN	4824	50	2412	50	1206	1206
3	PKNN	9648	100	2412	50	1206	1206
4	BP	0	0	2412	50	1206	1206
5	RBF	0	0	2412	50	1206	1206
6	ELM	0	0	2412	80	1930	482
7	PKNN	9328	100	1458	80	1166	292

**Table 3:** Comparison of prediction accuracy for seven groups of training experimental settings.

Group	MRE for Test Sets (%)								Total Error (%)
	ΔRe(S11)	ΔIm(S11)	ΔRe(S12)	ΔIm(S12)	ΔRe(S21)	ΔIm(S21)	ΔRe(S22)	ΔIm(S22)	
1	1.27	2.88	1.22	3.84	4.17	4.01	4.25	5.13	3.35
2	0.96	1.39	1.12	2.98	1.82	1.37	2.59	1.97	1.78
3	0.16	0.62	0.38	1.86	1.28	0.40	1.10	1.04	0.86
4	2.59	8.07	9.69	13.36	0.95	1.31	3.84	2.25	5.26
5	2.55	3.27	10.43	19.52	0.23	1.17	1.96	6.24	5.67
6	1.04	1.40	0.76	2.73	1.94	2.29	3.72	2.38	2.01
7	0.51	0.38	0.32	1.47	0.67	0.47	0.98	1.09	0.74

**Table 4:** Comparison of optimization efficiency and convergence of the networks.

Comparison	NETWORK1	NETWORK2	NETWORK2’
Fitness range	2.03~2.38	2.11~2.84	7.30~9.55
Total duration of optimization (s)	1956	5845	3728
Number of iterations to reach convergence	864	845	583
Convergence time (s)	1690	4939	2173

## Data Availability

Not applicable.
